# Fungal Identification and Empirical Antifungal Treatment in Critically Ill Patients with Secondary Peritonitis—A Retrospective Cohort Study

**DOI:** 10.3390/antibiotics15090842

**Published:** 2026-08-31

**Authors:** Patrícia Bernardo, Susana M. Fernandes, Ana S. Nepomuceno, Denise Pinto, João M. Ribeiro, João Gouveia

**Affiliations:** 1 Clínica Universitária de Medicina Intensiva, Faculdade de Medicina, Universidade de Lisboa, Avenida Professor Egas Moniz, 1649-028 Lisboa, Portugal; patriciabernardo@campus.ul.pt (P.B.); anasofia.nepomuceno@gmail.com (A.S.N.); 2 Serviço de Medicina Intensiva, Unidade Local de Saúde de Santa Maria, Avenida Professor Egas Moniz, 1649-028 Lisboa, Portugal; denise.pinto@ulsba.min-saude.pt (D.P.); jmribeiro@ulssm.min-saude.pt (J.M.R.); gouveia.jl@gmail.com (J.G.)

**Keywords:** sepsis, secondary peritonitis, intra-abdominal candidiasis, *Candida* peritonitis, antifungal treatment

## Abstract

Background/Objectives: The clinical impact of empirical antifungal therapy in patients with secondary peritonitis and sepsis remains incompletely established. This study aimed to identify risk factors for fungal infection in this population and the impact of empirical antifungal therapy on clinical outcomes. Methods: This was a retrospective cohort study of critically ill patients who underwent abdominal surgery for visceral lesions. Demographic and microbiological data were extracted from electronic health records. Results: A total of 411 patients, 56.9% male and with an average age of 68.8 ± 15.8 years, admitted following urgent gastrointestinal surgery were included. Fungi were isolated from peritoneal fluid or blood in 13.9% of patients during their ICU stay, with a median hospital length of stay until identification of 10 days (interquartile range 1–19 days). *Candida albicans* was isolated in 63.2% of the positive samples. Empirical antifungal was provided in 27.3% (n = 112). Each additional surgical procedure (OR: 1.47; *p* < 0.001) was the only risk factor identified for fungal infection in multivariate logistic regression. ICU mortality among patients with and without fungal documentation was similar, as was ICU mortality among patients with and without empirical antifungal therapy. Conclusions: The mortality impact of fungal isolation remains uncertain. Our results suggest that routine empirical antifungal therapy should be approached with caution, prioritizing targeted therapy for patients with confirmed positive cultures or a persistent, refractory clinical course despite adequate source control. These findings should be interpreted as hypothesis-generating and warrant confirmation in future prospective studies before informing changes in clinical practice.

## 1. Introduction

Complicated intra-abdominal infections (IAI), including secondary and tertiary peritonitis, results from visceral organ injury, which may be spontaneous, traumatic, or a complication of elective abdominal surgery [1,2] Secondary peritonitis is the most common in clinical practice [3], with high mortality rates (between 20 and 60%), when associated with septic shock and multiple organ failure [4,5,6,7,8]. Intra-abdominal candidiasis (IAC) occurs in approximately 30 to 40% of critically ill surgical patients with secondary peritonitis [9,10]. It includes *Candida* peritonitis, which is described as an independent risk factor for mortality in Intensive Care Unit (ICU) patients [11]. Secondary peritonitis with sepsis and fungal identification continues to be a highly critical condition, carrying a mortality rate between 25% and 60% [11,12,13], and in most cases, it occurs after a surgical complication such as anastomosis leakage [14]. The diagnosis of fungal peritonitis is often a late event, and there are no clear risk factors for the rational use of antifungal agents [10,14,15]. Eggimann et al. [16] evaluated the impact of prophylactic antifungal therapy, suggesting that its use decreased the incidence of *Candida* peritonitis in high-risk surgical patients. In a small retrospective study, empirical fluconazole was associated with a better prognosis among patients undergoing gastrointestinal surgery with occult *Candida* infection [17]. When associated with candidemia, early empirical antifungal therapy is associated with better outcomes across several heterogeneous cohorts, which included patients with risk factors other than abdominal surgery [18]. However, in several cohort studies, a positive impact of empirical antifungal therapy on morbidity and mortality among patients with fungal peritonitis has not been demonstrated, and international recommendations are therefore ambiguous regarding its use [13,15]. According to the most recent guidelines, empiric antifungal therapy should be considered for all patients with clinical evidence of IAI and at least one significant risk factor for candidiasis, including recent abdominal surgery, anastomosis dehiscence or necrotizing pancreatitis [19]. Nevertheless, this might lead to antifungal overuse. During the last decades, several clinical predictive scores for fungal infection have been developed in surgical and non-surgical patients. However, indications for empirical antifungal therapy in critically ill and surgical patients remain controversial, and there is still no precise score to predict fungal IAI [20,21]. Therefore, we aimed to evaluate the main risk factors for fungal infection in a real-world cohort of critically ill patients with secondary peritonitis admitted to the ICU and to characterize the use of empirical antifungal therapy and its putative association with outcomes in these patients.

## 2. Results

### 2.1. General Description

**Figure 1:**
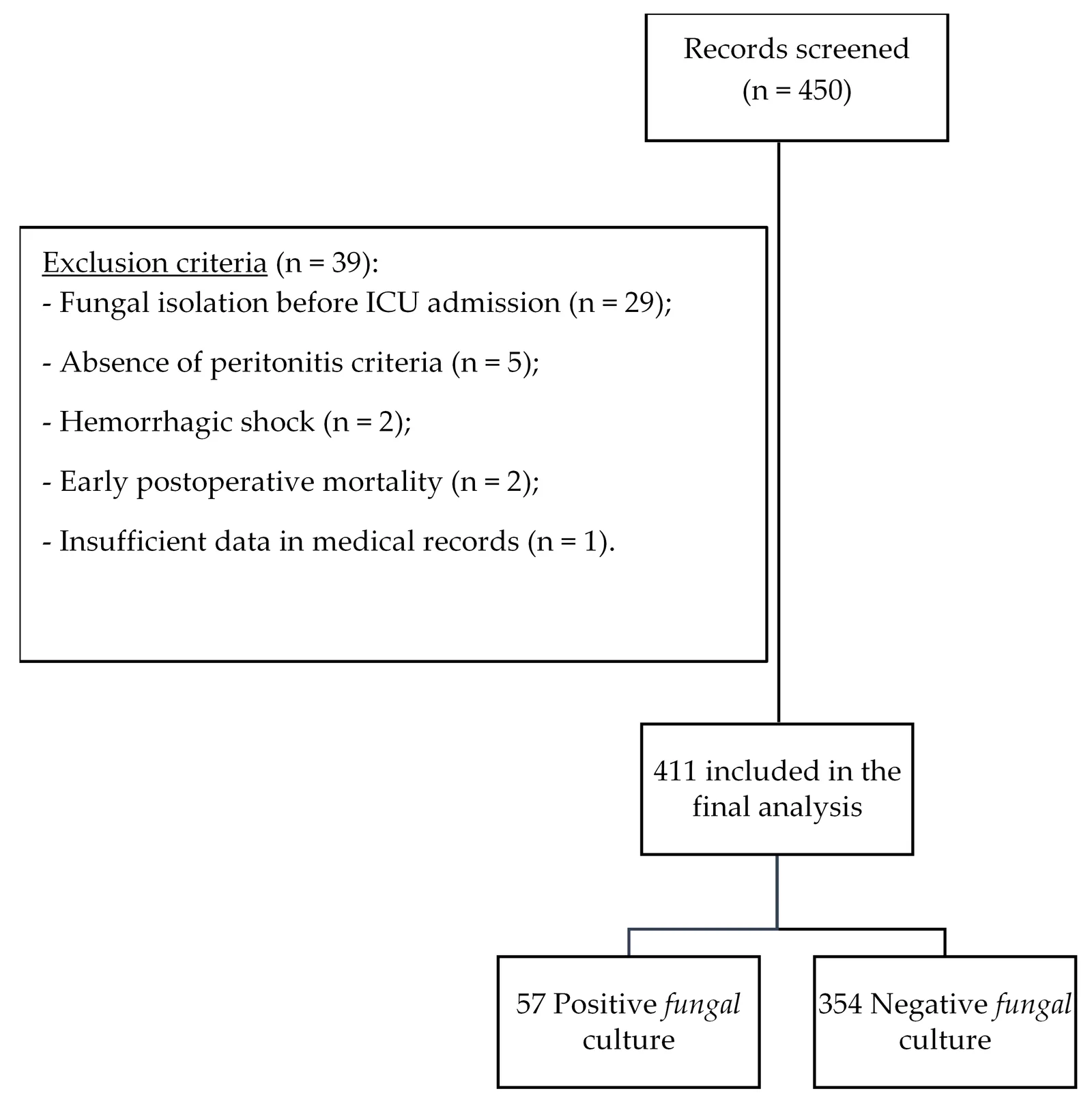
Flowchart for selection of study patients.

The study screened 450 ICU admissions following urgent abdominal surgery. After review of medical records, 39 patients were excluded due to fungal isolation before ICU admission (n = 29), absence of peritonitis criteria (n = 5), hemorrhagic shock (n = 2), early postoperative mortality (n = 2), and insufficient data in medical records (n = 1). In total, 411 admissions were included in the final analysis (Figure 1).

The study population included 234 men (56.9%) with a mean age of 68.8 years (±15.8). The mean Simplified Acute Physiology Score (SAPS) II was 50.4 (±18.8), and the mean Sequential Organ Failure Assessment (SOFA) at admission was 7.6 (±4.4), with 48.4% (n = 199) of the patients featuring septic shock. The median hospital length of stay (LOS) until the surgery leading to ICU admission was 1.0 day (interquartile (IQ) range 0–2, with a maximum of 38 days), and the median hospital LOS until ICU admission was 2.2 days (IQ range 1.1–8.7).

### 2.2. Risk Factors for Fungal Isolation

Positive fungal culture associated with ICU admission was found in 57 patients (13.9%). In eight additional patients, cultures were later positive for fungi in cultures not associated with ICU admission (from 17 to 78 days after ICU admission). The clinical and demographic characteristics of patients with and without fungal isolation associated with critical illness are described in Table 1. Among the isolated fungi, *Candida albicans* was the most prevalent (63.2%), followed by *Candida glabrata* (15.8%). Other non-albicans species were also identified: *Candida lusitaniae* (5.3%), *Candida krusei* (5.3%), *Candida tropicalis* (3.5%), *Candida parapsilosis* (1.8%) and *Candida famata* (1.8%). In one patient (1.8%), two *Candida* species (*lusitaniae* and *krusei*) were identified, and in another, no species was identified (1.8%).

The median hospital LOS until fungal isolation was 10.0 days (IQ range 1–19; minimum 0 and maximum 46 days), while the median ICU hospitalization time until the microbiological result confirming fungal isolation was available was 1.4 days (IQ range 0–6).

Microbiological identification was obtained intraoperatively from peritoneal fluid, except in seven patients with candidemia. There was no major difference in age, comorbidities, or the number of surgical procedures before fungemia compared with patients with only positive peritoneal samples. Of note, the median hospital LOS before a positive blood culture was tendentially longer (10 vs. 28 days, *p* = 0.06).

In the univariate analysis, the severity scores (SAPS II and SOFA) were not associated with a higher likelihood for fungal isolation, nor was septic shock (OR: 1.21 (95% CI 0.69–2.13); *p* = 0.49). However, each additional gastrointestinal surgical procedure increased the risk of fungal isolation (OR: 1.58 (95% CI 1.27–1.96); *p* < 0.01). Also, patients with anastomotic leak (OR: 1.87 (95% CI 1.04–3.36); *p* = 0.04) and those receiving parenteral nutrition (OR: 2.25 (95% CI 1.04–4.92); *p* = 0.04) had a higher risk of fungal infection (Table 2).

These variables and severity were included in a multivariate analysis. As community-acquired peritonitis and anastomotic leak overlap in more than 90%, we maintained only anastomotic leak in the multivariate analysis. Only the number of additional surgical procedures was associated with an increased risk of fungal isolation (OR: 1.47 (95% CI 1.11–1.96); *p* < 0.001). The remaining variables were no longer associated with fungal isolation (Table 3).

### 2.3. Antifungal Empirical Therapy

Empiric antifungal therapy was administered to 27.3% of patients (n = 112), of whom 26 subsequently had fungal isolation confirmed. Thus, 31 patients with fungal isolation did not receive empirical antifungal therapy. The most common antifungal used was fluconazole (72.3% of patients, n = 81), followed by anidulafungin in 11.6% (n = 13). Compared with patients who did not receive antifungal therapy, those who did were younger (67 vs. 73 years, *p* < 0.01), had lower severity scores (SAPS II 47 vs. 51, *p* = 0.03), and more frequently had undergone two or more surgical procedures by the time of ICU admission (22.8% vs. 12.5%, *p* = 0.01). The empirical administration of antifungal agents did not impact mortality, even when adjusted for severity (OR: 1.6; *p* = 0.130) or for fungal isolation on culture (OR: 1.06; *p* = 0.821). Of note, patients with no fungal isolation were exposed to antifungals for a median of only 2 days (IQ range 1–4), suggesting that antifungal therapy was stopped as soon as culture results were available.

### 2.4. Outcomes

In this study, fungal isolation was not associated with ICU mortality (19.3% among patients with positive cultures vs. 24.0% among those with negative cultures; *p* = 0.44), nor with hospital mortality (42.1% vs. 35.3%, *p* = 0.32), although the study was underpowered to identify absolute mortality differences greater than 19.6%. Notwithstanding this, patients with fungal isolation had increased ICU LOS, hospital LOS, and number of days on invasive mechanical ventilation (Table 4), reflecting also a longer hospital LOS before fungal identification and accumulated frailty.

## 3. Discussion

This study included a cohort of 411 critically ill adult patients diagnosed with peritonitis following urgent gastrointestinal surgery. Fungal isolation in the first 14 days after ICU admission was observed in 13.9%, and although not associated with higher mortality, it was associated with longer hospital stays and a longer duration of invasive mechanical ventilation.

The clinical relevance of fungal presence in peritoneal fluid during peritonitis remains debated. Recently, a study with a similar design associated the presence of *Candida* albicans in peritoneal swabs after gastric perforation with increased mortality [22]. However, it lacked information regarding the duration of clinical symptoms prior to swab collection or the presence of overt peritonitis following ulcer perforation. Peritonitis following ulcer perforation is initially predominantly chemical. Therefore, peritoneal detection of *Candida* might merely reflect a higher baseline gastric or esophageal colonization associated with immunosuppression, or simply the older age of the patients from whom *Candida* was isolated. Of note, in our cohort we did not find any association between gastrointestinal injury (upper or lower) and the presence of *Candida* spp. in the peritoneum.

In another study [3], female gender, upper gastrointestinal lesion, previous antibiotic therapy and perioperative shock were identified as important risk factors for fungal peritonitis. Neither age nor sex was a relevant factor in our study, but our patients were older than those in these studies, which might reduce the impact of age.

Beyond the study by Dupont et al. [3], other cohorts have examined risk factors for intra-abdominal candidiasis. The EUCANDICU case–control study, conducted across 26 European ICUs, identified recent abdominal surgery, anastomotic leak, and upper gastrointestinal perforation as independent risk factors [23]. A more recent single-center cohort of patients undergoing emergency gastrointestinal surgery for complicated IAI identified prior antibiotic exposure and multiple surgical interventions as independent predictors of invasive candidiasis [24]; the latter closely mirrors our study, in which each additional surgical procedure increased the risk of fungal isolation. Other groups have also implicated corticosteroid exposure, mechanical ventilation, and total parenteral nutrition as contributing risk factors [25]. Taken together, these studies support surgical burden and repeated abdominal interventions as a consistent risk factor across differing cohorts, even though individual predictive models vary considerably in the specific variables retained, likely reflecting differences in case-mix, definitions, and statistical approach.

The association we observed between fungal isolation and both prolonged invasive mechanical ventilation and hospital LOS is consistent with, rather than novel to, previous reports. A recent cross-sectional study in a surgical ICU similarly found that *Candida* isolation was associated with a substantially longer duration of mechanical ventilation than in matched controls, as well as higher mortality [26]. Our findings corroborate this association, reinforcing the interpretation that fungal isolation is more likely a marker of a complicated and protracted ICU course than an independent driver of outcome.

It is still controversial whether to universally apply empirical antifungal treatment in the management of patients with complicated intra-abdominal infection, especially those not presenting with septic shock. Importantly, recent data from a European study addressing mortality in critically ill patients found that while urgent source control was associated with improved odds of survival, the appropriateness of empirical antimicrobial treatment was not [27].

One important reason for the limited efficacy of antifungal drugs in several studies might be their poor penetration into the peritoneal fluid, resulting in subtherapeutic concentrations. This not only compromises treatment efficacy but also promotes antimicrobial resistance by facilitating rapid fungal escape [28].

Consistent with this and other cohorts [29], our retrospective analysis observed no survival benefit associated with empirical antifungal therapy, even after adjusting for baseline severity and fungal isolation. While the retrospective design limits our ability to draw causal conclusions, these findings align with growing concerns that fungal overtreatment may contribute to antimicrobial resistance and the emergence of more virulent non-*albicans Candida* species. This supports the hypothesis that, in many critically ill patients, a positive *Candida* culture may act primarily as a marker of profound illness severity—a “bystander”—rather than the direct driver of clinical complications [13]. Consequently, routine empirical antifungal therapy should be approached with caution. Until prospective data offer clearer guidance, it may be considered to limit its use, prioritizing targeted therapy for patients with confirmed positive cultures or refractory cases of tertiary peritonitis.

An important methodological caveat of our study concerns the non-random allocation of empirical antifungal therapy. Patients who received empirical antifungal therapy differed systematically from those who did not: they were younger, had lower severity scores, and were more likely to have undergone two or more surgical procedures by the time of ICU admission. This pattern suggests that treatment allocation was influenced both by accurate clinical recognition of surgical burden as a risk factor for fungal infection and by selection favoring younger, less severely ill patients. Notably, this selection would be expected to bias the comparison in favor of a survival benefit for antifungal therapy, given the more favorable baseline prognosis of the treated group; the absence of any observed benefit, despite this prognostic advantage, offers some reassurance that our null finding is not solely attributable to confounding by severity. Nonetheless, residual confounding by unmeasured factors cannot be excluded, and this observational design does not permit causal inference regarding the effectiveness of empirical antifungal therapy.

A related challenge is that fungal isolation from peritoneal fluid does not necessarily equate to invasive candidiasis. No universally accepted criteria currently exist to reliably distinguish true infection from colonization or contamination in this setting [15]. In our cohort, isolation in the context of confirmed intraoperative peritonitis was managed clinically as infection rather than contamination; nonetheless, we cannot exclude that in some patients—particularly those with prolonged antibiotic exposure or upper gastrointestinal lesions predisposing to mucosal fungal overgrowth—a positive culture reflected colonization rather than a true driver of sepsis. This uncertainty is consistent with the ‘bystander’ hypothesis discussed above and highlights the need for future studies to incorporate standardized criteria for distinguishing infection from colonization when defining fungal outcomes in this population.

An additional factor relevant to interpreting these findings is the duration of critical illness required before fungal infection becomes clinically relevant. Fungal isolation in our cohort occurred at a median hospital LOS of 10.0 days (IQR 1–19), and candidemia tended to occur even later than isolation from peritoneal fluid alone (median 28 days). Moreover, the only independent risk factor identified in our multivariate analysis (the number of additional surgical procedures) is itself a marker of a prolonged and complicated clinical course rather than a characteristic present at the time of the index emergency surgery. Taken together, these observations suggest that fungal infection should rarely be a primary concern at the time of the initial urgent surgery, before the patient has been exposed to the cumulative insults (repeated reinterventions, prolonged antibiotic exposure, parenteral nutrition, persistent failure of source control) that appear to drive IAC in this population. Rather than a blanket empirical strategy applied at the outset, antifungal coverage may be more rationally reserved for patients whose clinical course extends beyond the first several days, particularly those requiring further surgical reintervention or exhibiting persistent sepsis despite adequate source control. This time-dependent framework, prioritizing the duration and complexity of the critical illness trajectory over immediate empirical coverage, may help refine patient selection for antifungal therapy and merits prospective validation.

Several other limitations must be acknowledged. First, the single-center, retrospective design and the small subset of patients with fungal isolation in peritoneal fluid or blood limit the statistical power of our analyses (our sample is only powered to detect absolute differences in mortality above 19.6%). Moreover, the events-per-variable ratio for the multivariate model was approximately 9.5, at the conventional threshold for stable estimates, and forward selection may further increase the risk of overfitting.

In addition, we did not formally classify or track secondary versus tertiary peritonitis as distinct categories in our database; it is plausible that some patients progressed to tertiary peritonitis during a prolonged ICU course, but our data do not allow this to be quantified [30]. Furthermore, the retrospective nature of the study inherently introduces the potential for missing data and information bias. It should also be noted that the interval between data collection and the publication of this manuscript was longer than is typical, reflecting both the disruption of research activities during the COVID-19 pandemic and subsequent changes in institutional affiliation among several co-authors. While this delay does not affect the validity of the data collected, the reference guidelines on the diagnosis and management of candidiasis have since been updated [19]. While the qualitative ambiguity surrounding the indication for empirical antifungal therapy in secondary peritonitis persists, this should be borne in mind when considering the present-day applicability of our findings. While large prospective cohort studies remain indispensable to definitively establish the indications for empirical antifungal therapy, our findings offer valuable hypothesis-generating insights to be evaluated in other cohorts.

Overall, these findings are hypothesis-generating and warrant confirmation in future prospective studies; in the meantime, they may help inform local antifungal stewardship discussions.

## 4. Materials and Methods

### 4.1. Patient Selection

This was a retrospective study including all critically ill adult patients with secondary peritonitis [31], who underwent urgent gastrointestinal surgery and were admitted to the intensive care unit (ICU) of a tertiary university hospital between January 2013 and April 2018. Secondary peritonitis was defined as peritoneal inflammation resulting from direct contamination by spillage from the gastrointestinal tract. Patients were not formally classified or tracked according to progression to tertiary peritonitis during their ICU stay [30]. The diagnosis of peritonitis was based on the surgeon’s description of macroscopic findings during the operation. Patients were initially identified by querying the ICU administrative database for all urgent surgical admissions, followed by a secondary review of electronic medical records to confirm the presence of a visceral lesion associated with peritonitis. Patients without intraoperative evidence of peritonitis were excluded. Patients with a confirmed fungal infection prior to ICU admission were also excluded.

### 4.2. Data Collection

For each patient, demographics, primary diagnosis, organ support, main visceral lesion, antibiotic use, need for subsequent surgical interventions, ICU and hospital LOS were extracted from the electronic health record (EHR).

The study primary outcome was intra-abdominal fungal infection. This was defined as the presence of fungi in cultures (blood, peritoneal swabs or biopsies) and evidence of peritonitis as identified by the surgeon.

Secondary outcomes included ICU and hospital mortality.

Data were pseudo-anonymized and collected into an Excel-based database. The ethical committee of Centro Académico de Medicina de Lisboa approved the study on the 20 May 2024 (internal reference number 6/24). Informed consent was waived, given the retrospective nature of the study.

### 4.3. Microbiology and Antifungal Therapy

Fungal culture results were obtained by reviewing the microbiology laboratory database. Following urgent gastrointestinal surgery, patients were admitted to the ICU. A positive culture was defined as the isolation of a fungus from peritoneal fluid or blood, using standard culture methods concomitant with surgical evidence of infection. Only infections associated with ICU admission (until 14 days after ICU admission) were considered. Empirical antifungal therapy was evaluated; consequently, only antifungal therapy administered to treat an ongoing peritoneal infection prior to any culture results was evaluated. Empirical antifungal treatment was standard of care at the ICU for patients admitted with peritonitis and upper gastrointestinal lesions, but it is not universally applied to all postoperative patients with complicated intra-abdominal infections. All patients received concomitant antibacterial treatment.

### 4.4. Statistical Analysis

As appropriate, a descriptive statistical analysis of the database was conducted, followed by a comparative study using the *t*-test or Chi-squared test, or the Mann–Whitney U test for comparing medians. Univariate and multivariate logistic regression were performed using the forward-selection method to identify the relevant variables for fungal isolation. Univariate and multivariate logistic regressions were also performed to identify variables related to hospital and ICU mortality. Fungal isolation was forced into the mortality model. The statistical analysis was performed with STATA/SE v15.1 (StataCorp LLC, College Station, TX, USA). The results are expressed as mean ± standard deviation or medians and confidence intervals, as appropriate. The odds ratio (OR) and 95% confidence interval (95% CI) were calculated. A *p*-value less than 0.05 was considered significant.

## 5. Conclusions

Fungal identification is frequent in secondary peritonitis, particularly in patients with multiple interventions and prolonged length of stay. While frequent, its impact on mortality remains uncertain. Our results raise the hypothesis that routine empirical antifungal therapy can be approached with caution, prioritizing targeted therapy for patients with confirmed positive cultures or refractory cases of tertiary peritonitis. Further studies are needed to assess the impact of empirical antifungal therapy in these patients.

## Figures and Tables

**Table 1:** Clinical and demographic patient characteristics according to fungal culture results.

	Positive *Fungal* Culture (n = 57)	Negative *Fungal* Culture (n = 354)	*p* Value
Age (years)	69.1 ± 12.8	68.8 ± 16.2	0.87
Male gender (%)	34 (59.6)	200 (56.5)	0.66
*Diabetes Mellitus* type II (%)	8 (14.0)	57 (16.1)	0.69
Organ failure and support			
Septic Shock (%)	30 (52.6)	169 (47.7)	0.47
Acute Kidney Injury, n (%)	35 (61.4)	228 (64.4)	0.66
ARDS, n (%)	3 (5.3)	22 (6.2)	0.78
SAPS II	49.1 ± 15.4	50.6 ± 19.4	0.59
SOFA at admission	6.9 ± 4.0	7.7 ± 4.5	0.23
Invasive Mechanical Ventilation, n (%)	51 (89.5)	285 (80.6)	0.10
Secondary peritonitis—culture and treatment			
Community-acquired peritonitis (%)	33 (57.9)	261 (73.8)	0.02
Anastomotic leak, n (%)	22 (38.6)	89 (25.1)	0.04
Broad-spectrum antibiotic, n (%)	49 (86.0)	271 (76.6)	0.11
Parenteral nutrition, n (%)	10 (17.5)	31 (8.8)	0.04
LOS in ICU when fungal result was obtained (days) *	1.4 (0–6)	N/A	-
Empirical antifungal therapy, n (%)	26 (45.6)	86 (24.3)	<0.01
Location of the abdominal lesion			0.07
Esophagus and stomach	14 (24.6)	54 (15.3)	
Duodenum	6 (10.5)	17 (4.8)	
Jejunum and ileum	15 (26.3)	97 (27.4)	
Colon and rectum	18 (31.6)	129 (36.4)	
Gallbladder	4 (7.0)	57 (16.1)	
Fungal culture			
*Candida albicans*, n (%)	36 (63.2)	N/A	

The results are presented as mean ± standard deviation, percentage, or median and interquartile range. * calculated as day of admission in the ICU—day of positive result. N/A, Data not available. ARDS, Acute Respiratory Distress Syndrome; SAPS II, Simplified Acute Physiology Score II; SOFA, Sequential Organ Failure Assessment; ICU, Intensive Care Unit; LOS, Length of stay.

**Table 2:** Univariate analysis of risk factors for fungal isolation.

	OR	*p* Value	CI 95%
Male gender	1.14	0.66	0.64–2.01
SAPS II	0.99	0.59	0.98–1.01
Septic shock	1.21	0.49	0.69–2.13
Additional surgical procedures	1.58	<0.01	1.27–1.96
Anastomotic leak	1.87	0.04	1.04–3.36
Parenteral nutrition	2.25	0.04	1.04–4.92
Community acquired peritonitis	0.48	0.01	0.27–0.83

CI, confidence interval; SAPS, Simplified Acute Physiology Score; OR, odds ratio.

**Table 3:** Multivariate analysis of risk factors for fungal isolation.

	OR	*p* Value	CI 95%
Additional surgical procedures	1.47	<0.01	1.11–1.96
Anastomotic leak	1.15	0.693	0.57–2.32
Parenteral nutrition	1.04	0.936	0.39–2.77
SAPSII	1.00	0.966	0.98–1.01

CI, confidence interval; SAPS, Simplified Acute Physiology Score; OR, odds ratio.

**Table 4:** Outcome of patients according to peritoneal or blood fungal culture results.

	Positive *Fungal* Culture (n = 57)	Negative *Fungal* Culture (n = 354)	*p* Value
ICU associated Mortality (%)	19.3	24.0	0.44
Hospital associated mortality (%)	42.1	35.3	0.32
ICU LOS (days)	4.4 (1.9–9.1)	3.1 (1.6–5.8)	0.02
Hospital LOS (days)	36 (20–74)	17 (9–37)	<0.01
Invasive mechanical ventilation (days)	3 (2–5)	2 (1–4)	<0.01

LOS, Length of stay; Medians and interquartile ranges are shown. Comparisons for continuous variables were made using Mann–Whitney test. Mortality was compared using proportions.

## Data Availability

Data are contained within the manuscript. Raw data is available from the corresponding author upon reasonable request.

## Abbreviations

The following abbreviations are used in this manuscript:

EHR	Electronic Health Record
IAC	Intra-abdominal Candidiasis
IAI	Intra-abdominal Infection(s)
ICU	Intensive Care Unit
IQ	Interquartile
LOS	Length of stay
OR	Odds Ratio
SAPS II	Simplified Acute Physiology Score II
SOFA	Sequential Organ Failure Assessment
95CI	95% confidence interval
